# Supplementary material for: Transcriptome and Small-RNA Sequencing Reveals the Response Mechanism of Brassica napus to Waterlogging Stress
Source: Plants (Basel). 2025 Apr 29;14(9):1340. doi: 10.3390/plants14091340 (PMC12073736; doi:10.3390/plants14091340)
Supplement: Supplementary file 1 [file plants-14-01340-s001.zip › Supplementary figures.pdf]

Supplement Figures

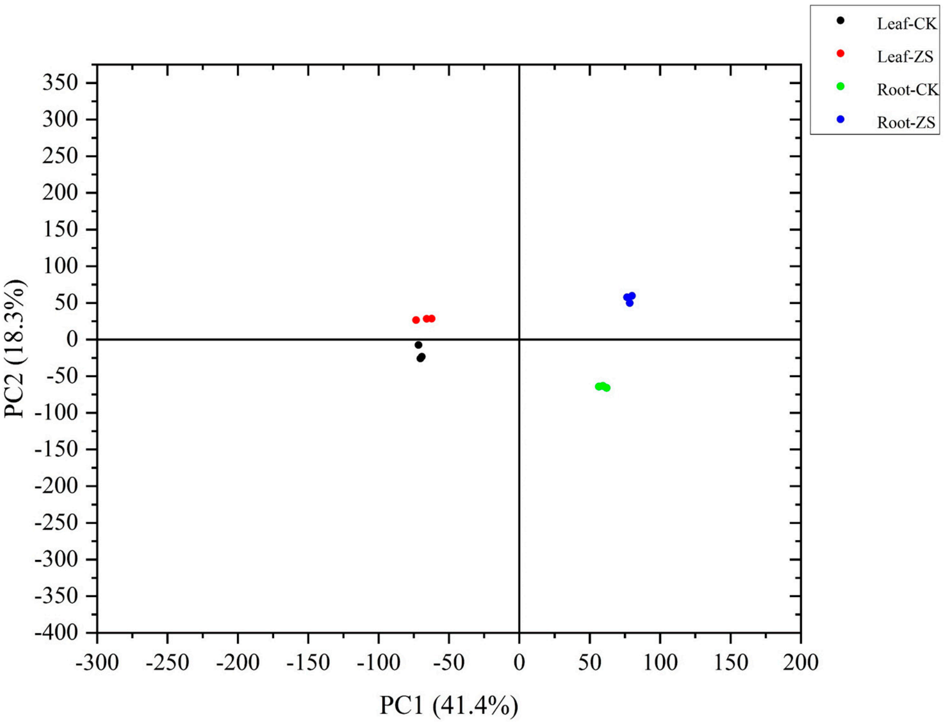

Figure S1. PCA analysis based on the transcriptome data.

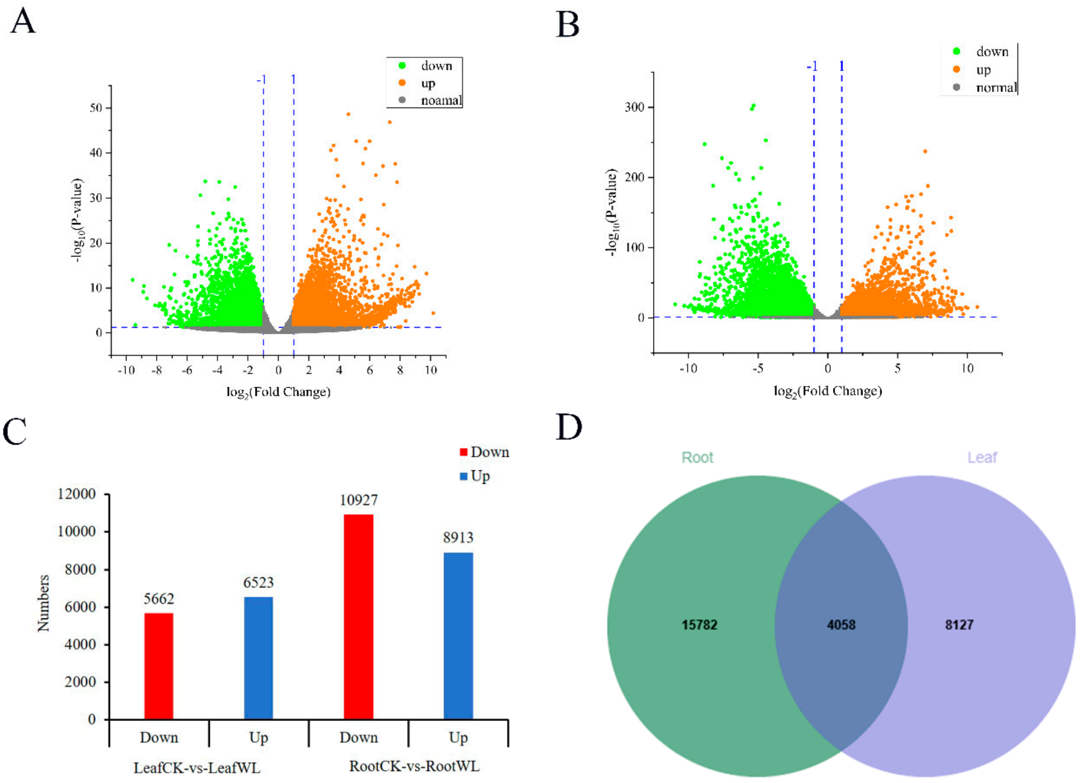

Figure S2. DEGs identified between the CKT and WLT. (A) Volcano plot of DEGs identified in leaves; (B) Volcano plot of DEGs identified in roots; (C) The expression profiles of waterlogging-induced DEGs in the roots and leaves. (D) Shared and unique DEGs between leaves and roots.

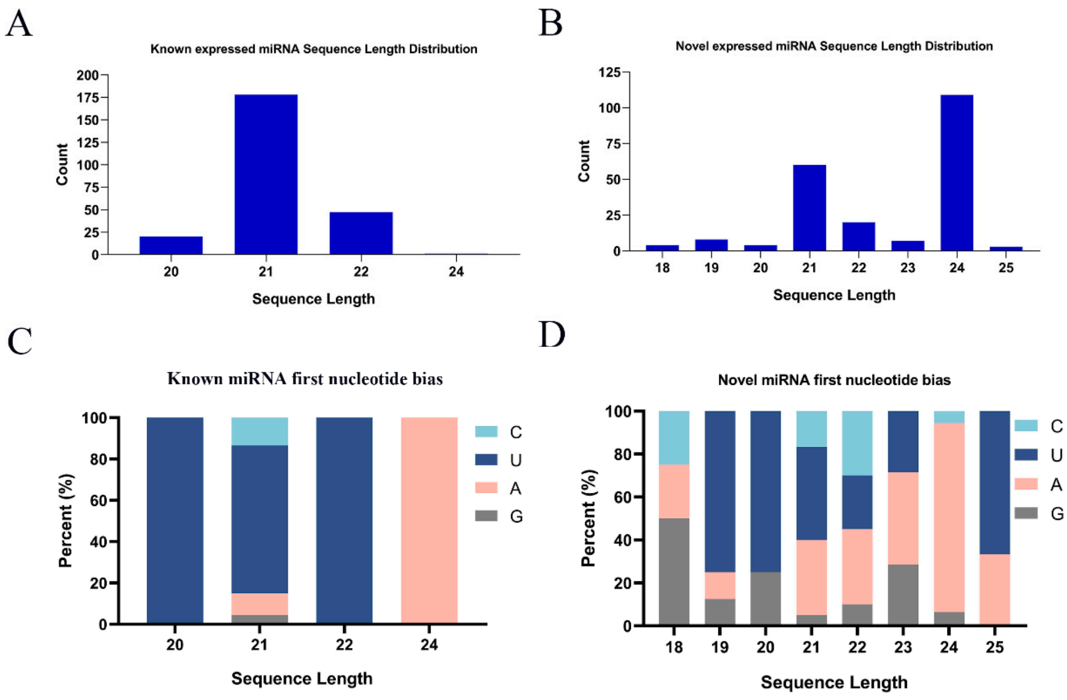

Figure S3. Structural characteristics of miRNA predicted in leaves. (A) Statistics of known miRNA sequence lengths; (B) Statistics of novel predicted miRNA sequence lengths; (C) Distribution map of the first base of known miRNAs of different lengths; (D) Distribution map of the first base of novel predicted miRNAs of different lengths.

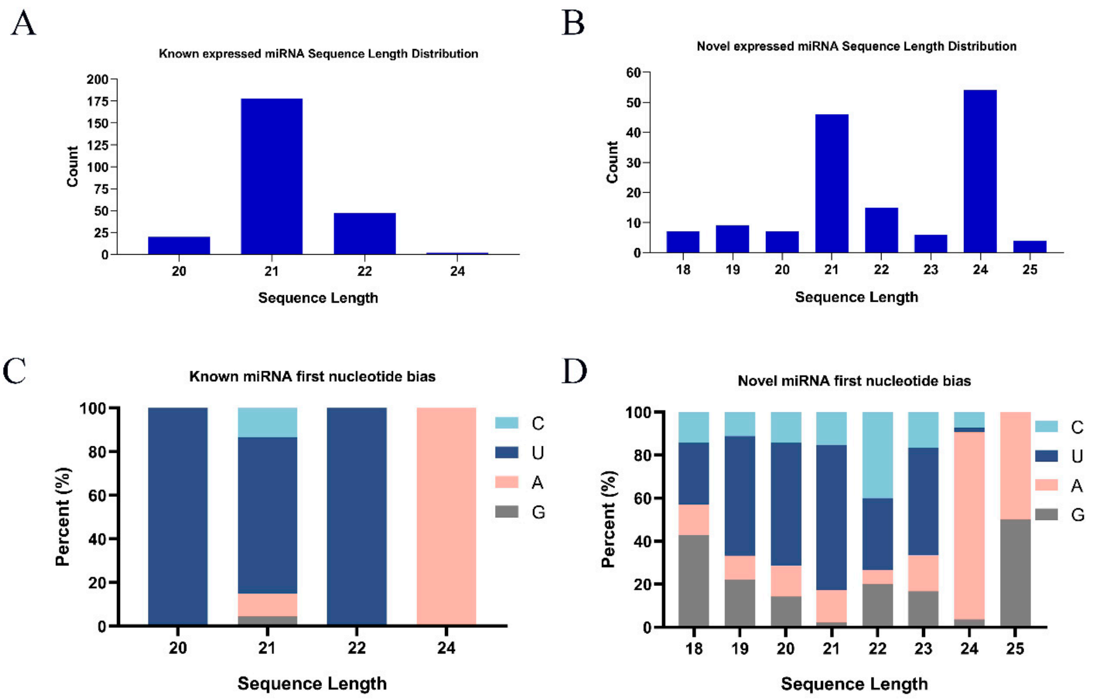

Figure S4. Structural characteristics of miRNA predicted in roots. (A) Statistics of known miRNA sequence lengths; (B) Statistics of novel predicted miRNA sequence lengths; (C) Distribution map of the first base of known miRNAs with different lengths; (D) Distribution map of the first base of novel predicted miRNAs with different lengths.

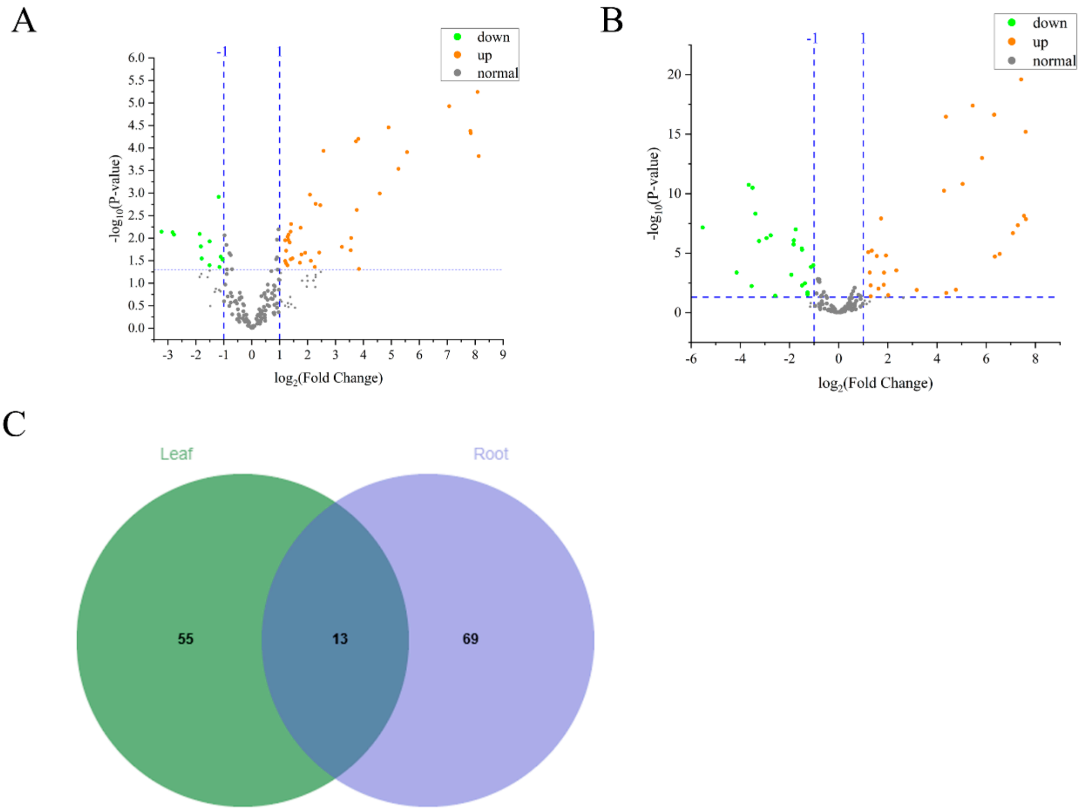

Figure S5. DEmiRNAs identified in leaves and roots. (A) Volcano plot of DEmiRNAs identified in leaves. (B) Volcano plot of DEmiRNAs identified in roots. (C) Shared and unique DEmiRNA families between leaves and roots.
